# Evaluating the Effectiveness of a Virtual Simulation Platform for Apexification Learning

**DOI:** 10.3390/dj12020027

**Published:** 2024-01-29

**Authors:** Lan Ma, Hongbin Lai, Wei Zhao

**Affiliations:** Department of Pediatric Dentistry, Guanghua School of Stomatology, Hospital of Stomatology, Sun Yat-Sen University, Guangzhou 510275, China; malan6@mail.sysu.edu.cn (L.M.); laihb3@mail2.sysu.edu.cn (H.L.)

**Keywords:** virtual simulation, undergraduate dental education, apexification, education methodology

## Abstract

The traditional teaching methods for apexification face difficulties in meeting dental students’ practical training needs. Herein, we aimed to find optimal techniques of learning apexification and to evaluate whether a virtual simulation platform for apexification learning was effective. A virtual simulation learning platform for apexification was developed. Ninety-nine 4th-year dentistry students were classified randomly into the control group (Con, receiving conventional teaching) and the experimental group (Exp, receiving virtual simulation teaching). Theoretical tests before (test 1) and after the virtual simulation training (test 2) assessed the effect of learning. In the Exp group, a questionnaire was used to assess student understanding of the virtual simulation platform. In the Exp group, the test-2 scores were significantly better compared those in the Con group (*p* < 0.001). Furthermore, in the Exp group, the test-2 scores exceeded those of test 1 (*p* < 0.001). Feedback using the questionnaire covered the evaluation of the Exp group for the virtual reality platform and offered useful suggestions. Applying the virtual simulation learning platform had positive effects on improving learning quality related to apexification.

## 1. Introduction

Digital technologies, including artificial intelligence, big data, virtual reality, and others, have rapidly developed in the recent years and play an increasingly vital role in the evolution of the whole society, as well as in the education area. Recently, a new model of combining virtual simulation technology with education has been applied in many aspects. Virtual simulation (VS) education refers to the involvement of virtual reality, augmented reality, mixed reality, or screen-based platforms [1]. Basically, VS education depends on the interaction between participants and computer-generated simulations to make learning content more attractive and enhance learning motivation. In brief, the application of VS technology in pedagogy allows operators to experience three-dimensional scenes similar to reality generated by the computer, gaining a better understanding of related information [2,3]. Moreover, VS education overcomes the limitation of spatial and physical requirements, which makes it a great tool for remote teaching and training during the COVID-19 pandemic [4].

Compared with traditional education methods, virtual simulation education aims to explore more personalized, intelligent, and ubiquitous pedagogical models. The objective is to build an open and shared information-based teaching demonstration system with a professional layout and excellent teaching effect, aiming to comprehensively enhance the quality of education in higher education [5]. The application of VS technology in the dentistry has become widespread. In China, the opening of the utilization of virtual simulation in dental education started around ten years ago, and now an integral virtual simulation teaching system has been constructed and incorporated into various teaching elements in some dental schools [6]. The advantages of VS technology in teaching dental profession courses have been gradually realized and accepted by teachers and students, followed by the reformation of teaching effects [7]. In 2019, the Ministry of Education of the People’s Republic of China started a project to establish near 1500 first-rate VS experimental courses at the end of 2021, showing their ambitions for VS education.

Clinical skills are an indispensability part of dentistry, and VS experimental platforms have become especially popular in recent years. For example, Murbay et al. reported that the performance of undergraduate dental students in the pre-clinical operative dentistry course could be greatly improved after exposure to the Simodont^®^ dental trainer [8]. A virtual simulation platform combined with a jaw simulation model has been proven to enhance students’ implantology skills and training, thus improving the quality of oral implant education [9]. The evaluation of various dental simulators for education in endodontics, periodontics, oral and maxillofacial surgery, dental radiography, prosthodontics, implantology, and orthodontics has been reviewed [10].

Pediatric dentistry deals with children from birth through adolescence. However, dental students often find it stressful to treat pediatric patients because young patients fail to cooperate well during complex or lengthy treatments. Pediatric dentists usually need to spend time comforting the patient by practicing appropriate behavior management, which might reduce the time required to finish common dental procedures. Therefore, it is essential to enhance the educational quality of pediatric dentistry courses to improve students’ clinical skills. For this purpose, a virtual simulation platform is a good choice. Zafar et al. demonstrated that the Simodont^®^ dental trainer could enhance the training of dental students with regard to restorative exercises for pre-clinical pediatric dentistry [11]. Another study proved that the application of dental local anesthesia virtual simulation can improve the engagement and learning experience of students in pediatric dentistry [12]. Lu et al. constructed an experimental VS teaching platform for vital pulpotomy, which could effectively enhance pulpotomy teaching [13]. Philip et al. implemented a pilot pedagogical study to assess the effectiveness and student perceptions of haptic virtual reality simulation devices as teaching tools in pre-clinical pediatric dentistry, but no significant differences were found between the experiment and control groups [14]. However, VS platforms are still rarely used in the field of pediatric dentistry.

When trauma or caries affects an immature tooth, its vitality and levels of inflammation determine the involvement of the pulp. Pulp necrosis or irreversible inflammation disturbs root-end closure procedures of immature permanent teeth to develop a fully formed apex. The dental treatment termed apexification is performed to remove the necrotic pulp from immature permanent teeth, to effectively perform root canal disinfection, and to obturate the root canals with calcium hydroxide, mineral trioxide aggregate (MTA), or other root repair materials with good biocompatibility, which will induce a calcified barrier at the apex of the root [15]. Lu et al. reported a retrospective study where apexification with modified calcium hydroxide paste achieved good outcomes after treating nonvital immature permanent teeth [16]. Shaik et al. performed a meta-analysis to compare the success rate of MTA, a bio-ceramic root repair material, and calcium hydroxide for the apexification of necrotic immature permanent teeth and found that all three of these biomaterials showed similar success rates in terms of clinical symptoms, but calcium hydroxide for apexification requires more time for apical barrier formation and multiple appointments [17]. Apexification turns out to be a cost-effective choice compared to regenerative endodontic treatment in the treatment of necrotic immature permanent teeth [18]. However, this technique requires complicated clinical skills and several monthly appointments, resulting in a high failure rate among students [19]. Unfortunately, because of the limitations of expenditure, human material resources, and laboratory devices, students usually fail to master the technique of apexification prior to working in the clinic. Moreover, we lack a model of young permanent teeth with open apices, which restricts students’ learning opportunities.

Herein, to improve the learning of competence for the diagnosis and treatment of apexification, we independently created a simulated virtual learning platform, including the examination, diagnosis, treatment plan, operating steps, and prognosis of apexification (https://xnfz.sysu.edu.cn/vlab/gjydx.html, accessed on 10 October 2023). Furthermore, we evaluated the effectiveness of the virtual simulation platform for apexification learning through tests and questionnaires.

## 2. Materials and Methods

### 2.1. Basics of Apexification Learning Using the Simulated Virtual Platform

#### 2.1.1. General Information about the Virtual Simulation Learning Platform

The Beijing Rainer Network Technology Company (Beijing, China; www.rainier.net.cn, accessed on 10 October 2023) supported the developed learning platform. The platform comprises network technologies, multimedia elements, and computer simulation, which can be visited through this link: https://xnfz.sysu.edu.cn/vlab/gjydx.html (accessed on 10 October 2023).

**Figure 1:**
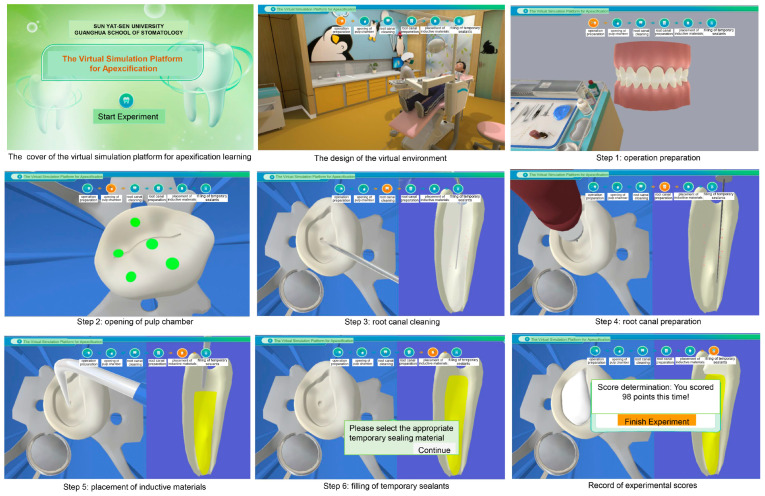
The virtual simulation platform for apexification learning.

The platform simulates the clinic environment in our hospital, comprising the clinic layout, the equipment, and medical materials. Moreover, the platform simulates the entire process from reception to examination, diagnosis, and treatment of immature permanent teeth subjected to fracture of dens evaginatus. In this VS situation, six key steps of clinical technology are covered, including operation preparation, opening of the pulp chamber, root canal cleaning, root canal preparation, placement of inductive materials, and filling with temporary sealants (Figure 1). Eleven single-choice questions are used to assess the standards and contents of objective evaluation based on the mastery of the vital skills required in clinical practice. At the same time, corresponding scores are given. Analyses of the answers to most questions are also presented after the correct answer is selected. Furthermore, the experiment is repeatable, and the analysis of human–computer interactions can be implemented retrospectively. Teachers can track the student’s learning status interactively via network learning. Moreover, users outside the campus of Sun Yat-Sen University can visit and use this platform. User access to the platform is unlimited.

#### 2.1.2. Details about the Simulated Virtual Learning Platform

After clicking on the button “starting the experiment”, users can enter into a simulated clinic room including a dentist, a child, and cute layouts similar to those in pediatric dentistry department. At the top of the page, six key steps are displayed and the ongoing step is marked by orange color. On the right side of the interface, all the medical materials and equipment are presented, which could be used for the treatment. The outline of all the questions presented in the virtual simulation platform has been displayed in the Appendix A, including the questions, the options, and the corresponding explanations. The whole process will be introduced in the following part.

**Figure 2:**
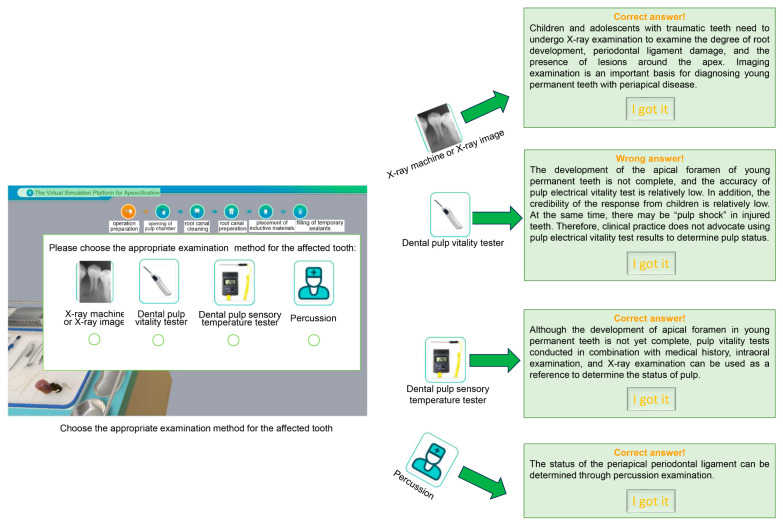
Schematic illustration of the questions presented in the virtual simulation platform.

In step 1 (operation preparation), the chief complaint of the simulated patient is shown, describing the type and degree of the pain and the position of the teeth. The clinical examination results are also shown. The first question concerns which examinations should be taken to clarify the diagnosis, and four options can be provided. If users choose the right answer, the interface moves to the next stage; otherwise, the page will go back to the original question and users have another chance to make their choice. The platform prepares explanations for each choice in this case. For the first question, there are three right answers and one wrong answers, as shown in Figure 2. Next, users need to choose the proper treatment methods for the simulated patient from four options, including pulpectomy, direct capping, apexification, and root canal treatment. In this simulated situation, apexification should be the best option, and explanations have also been given. After that, a question about devices isolating the treatment site from saliva, tongue, and cheek is asked, emphasizing the importance of rubber dam in clinical practice.

In step 2 (opening of pulp chamber), users need to choose the correct position from the provided dots in the occlusal surface of simulated virtual teeth to enter into pulp. Next, in order to remove the top of the pulp and establish a channel, users are asked to choose the types of dental handpieces (high-speed or slow-speed) and burs (fissure bur or round bur) from material panel at the right of the interface. Similarly, the related explanations are given.

In step 3, according to the general principal of root canal cleaning, users should choose the suitable root canal irrigating solutions after removing the necrotized pulp with pulp extraction needle. Several options have been provided, including 5.25% sodium hypochlorite (NaClO), 17% EDTA solution, 7.5% povidone–iodine solution, and the combined solution of 3% hydrogen peroxide solution and saline. The correct answer is only one. Each selection has the corresponding explanation. The whole process of root canal cleaning is simulated. After irrigating the root canal, absorbent paper points are used to make it dry.

In step 4 (root canal preparation), a single-choice question is about the proper nickel–titanium (NiTi) instruments for root canal preparation of immature permanent teeth. Users need to make a choice from the 8#, 15#, and 30# NiTi rotary files. The explanations for all right and wrong answers are given. The process of root canal preparation with NiTi files is implemented.

In step 5, users are firstly instructed to make preparations for placement of inductive materials: to clean the root canal and dry it. After the simulated preparations have been finished, users are asked to choose appropriate inductive materials for apexification from gutta percha point, Vitapex or Metapex, light cured calcium hydroxide, and conventional calcium hydroxide. The question is of single-choice type, and the reason for each option is illustrated in detail. Then, the simulated operations for placing inductive materials are shown. Taking X-ray is also required to examine the therapeutic effects.

In step 6, suitable temporary sealing materials need to be selected, between zinc oxide (ZOE) and glass ionomer, at the right material panel for sealing the teeth. Similarly, an explanation is also given when choosing the wrong answer. Next, users are asked to select a proper time for restoring the shape of the damaged teeth. Is it the time after finishing apexification or the time after closure of open apex? The reasons are given for the selection. Furthermore, the right time for permanent root canal therapy of the injured teeth with apexification is examined. The options include when the root apex of the affected tooth should be closed—at the age of 12 or at the age of 18. All answers have the related explanations.

At the end of the simulated virtual experiment, scores are given and recorded. Users can click the “homepage” button at the top right corner to return to the start page of this platform.

### 2.2. Study Procedure

**Figure 3:**
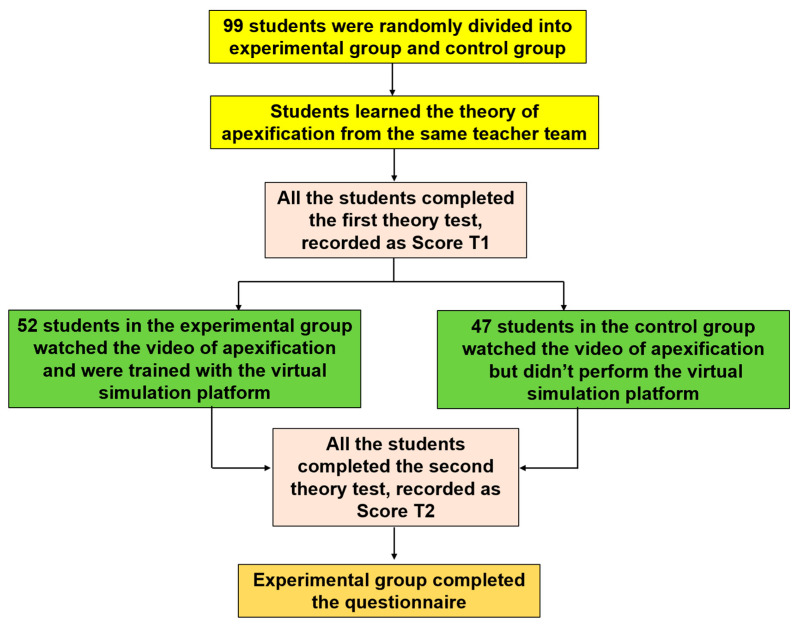
Schematic diagram of experimental design.

Upon review and confirmation of the submission documents, this project met the conditions for exemption from review by the Ethics Committee of the School of Stomatology, Sun Yat-Sen University (Institutional Review Board no. KQEC-2023-49-02). It was conducted involving the 4th-year dentistry students attending Guanghua School of Stomatology, Sun Yat-Sen University, from September 2022 to June 2023. A total of 106 students consented to participate and were classified randomly into the control group (Con, *n* = 53) and the experimental group (Exp, *n* = 53) using a random-number table, which can randomly draw the samples scientifically. Finally, 99 students participated and completed this project. The Exp group comprised 52 students and the Con group comprised 47 students. None of the subjects had received apexification courses before this study. The Con group received conventional teaching model. Students took a 2-teaching-hour theoretical course about apexification and were required to watch an apexification instruction video during experimental teaching. Instead, students of the Exp group learned the same theoretical course from the same teacher team and were asked to watch an apexification instruction video and perform virtual simulation platform training in the experimental class. The design of this study is shown in Figure 3.

Two tests (test 1 and test 2) for objective assessment were designed by three pediatric dentistry teachers with over 5 years of teaching experience and corrected by the professor of Department of Pediatric Dentistry. The contents of two tests can be reviewed (Appendix B and Appendix C). Each test contained 12 single-choice questions divided into three equal parts, covering diagnosis and prognosis (part A), surgical points of apexification (part B), and related knowledge expansion (part C). A correct answer to each question scored one point. The scores were assigned by the same teachers. After finishing the theoretical course, the Exp and Con groups completed test 1 (test 1, T1), the results of which were recorded as Score T1 (S_T1_). Both groups were required to watch the apexification video. Only the Exp group used the virtual simulation platform of apexification and finished the virtual simulation course. A week later, students in both groups completed test 2 (test 2, T2), based on the Ebbinghaus Forgetting Curve, and their T2 points were recorded as Score T2 (S_T2_).

Herein, the questionnaire used for the simulated virtual platform was in consistent with that used by Lu in 2022 [13]. It was designed by the teachers in the Department of Pediatric Dentistry affiliated with our university with the help of Survey Star platform (Ranxing Information Technology Co., Ltd., Changsha, China). The questionnaire contained 7 items. The first four items referred to the learning effects of this platform while the other three items were about the evaluation provided by students. Moreover, comments and advice from students about this platform were also collected. After test 2, the Exp group was asked to complete the questionnaire. The tests and questionnaires were completed in class, with a 100% recovery rate.

### 2.3. Statistical Analysis

SPSS version 20.0 software (IBM Corp., Armonk, NY, USA) was used to analyze the study data, the distribution of which appeared as the median, first, and third quartiles. The Shapiro–Wilk test was used to analyze the normality of data distribution. Comparisons between two groups were analyzed using the Mann–Whitney U test. Statistical significance was accepted at a *p* value less than 0.05. Graph analysis was performed using GraphPad Prism 8.00 (GraphPad Software, Boston, MA, USA).

## 3. Results

### 3.1. Score Comparison between the Con and Exp Groups

For test 1, the Exp and Con groups achieved similar total point scores (Table 1). Moreover, there was no significant difference in the scores of the three parts between the two groups (Table 1). These results indicated that students in the Con and Exp groups were at the same level in the diagnosis and prognosis of diseases, surgical points of apexification, and related knowledge expansion prior to the experiment, which provided a reliable baseline for comparison with the experimental intervention. After virtual simulated platform training on apexification, the Exp group had significantly higher total points in terms of S_T2_ than the Con group (*p* < 0.001) (Table 2). Interestingly, in test 2, the scores of parts A and C were not significantly different between the two groups whereas the Exp group had a significantly higher S_T2_ for part B (about surgical points of apexification) than the Con group (*p* < 0.001) (Table 2). Thus, the Exp group had mastered the operative knowledge of apexification better than the Con group.

### 3.2. Score Comparison in the Con and Exp Groups Separately

In the Con group, significantly higher total points (*p* < 0.05) and part-C points (*p* < 0.001) were gained in test 2 than in test 1 (Table 3), indicating that watching the video on apexification might have a positive effect in inspiring students to acquire more knowledge about apexification. The points of part A and part B in the Con group showed no significant difference (Table 3), which suggested that watching the video on apexification cannot improve the mastery of knowledge about diagnosis, prognosis, and operation skills. The Exp group had significantly higher total points for test 2 compared with those for test 1 (*p* < 0.001) (Table 4). In the Exp group, the score for part B was significantly different between test 1 and test 2 (*p* < 0.001) (Table 4), indicating that the VS learning platform for apexification enhanced the students’ mastery of the key operative points. Interestingly, the part-C points of test 2 in the Exp group were also significantly higher than those of test 1 (*p* < 0.001) (Table 4), but it was hard to make a conclusion about the contributing factors from watching the video, the VS platform, or both. Similarly, in the Exp group, the points for part A in test 2 had no significant difference compared with those in test 1 (Table 4).

### 3.3. Exp Group’s Responses to the Questionnaire

Questionnaire feedback from the Exp group (Table 5) indicated that 63.46% of students strongly agreed and 36.54% partially agreed that the VS learning platform was very helpful for learning the apexification technique. A total of 63.46% of students strongly agreed and 34.62% partially agreed that the VS learning platform improved the mastery of key points and the difficulties of apexification. All participants, strongly or partially, agreed that this platform was making it easier to become familiar with the apexification process. This educational method was liked, at least partially, by >90% of the participants, and they expressed increased enthusiasm for learning using simulation methods. Nearly 60% of the students thought that the VS platform was easy to operate, but over 30% of users felt it inconvenient and a waste of time.

**Figure 4:**
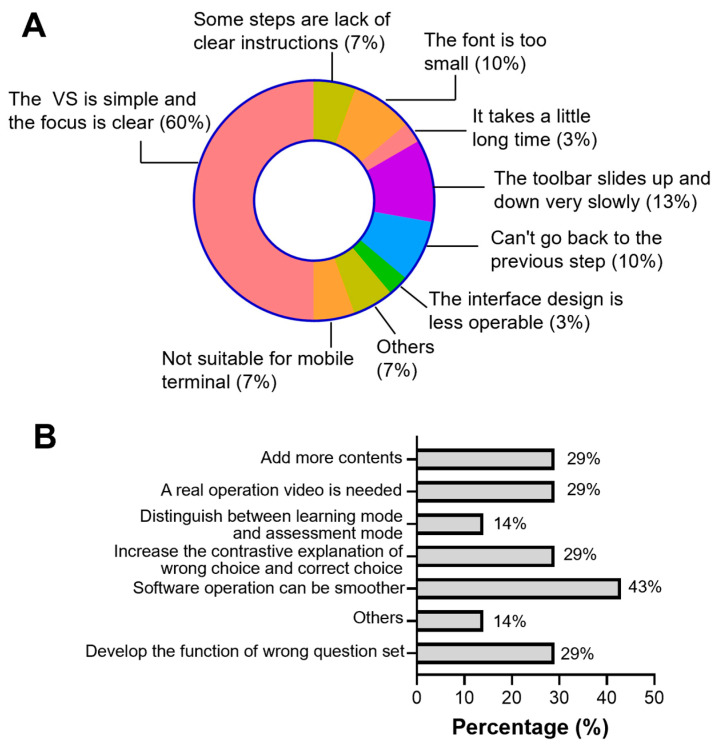
Graphical representation of the evaluation and improvement suggestions for the VR learning platform in the Exp group. (**A**) Evaluation of the VR learning platform. (**B**) Distribution analytics of the advice from the Exp group.

According to the evaluation given by the students, 60% of the participants thought that the VS platform was simple and the focus was clear. However, there are still some drawbacks that need to be improved, including the toolbar sliding up and down slowly (13%), the lack of a function for returning to the previous step (10%), the small font (10%), the lack of clear instructions in some steps (7%), inconvenience for mobile terminal visiting (7%), a slightly long time taken (3%), the less-operable interface design (3%), and others (7%) (Figure 4A). Students also offered some advice to improve this VS learning platform. They hope that the software operation can be smoother (43%) and more content can be added (29%). A real video about the operation process of apexification in the clinical is greatly expected (29%). They also suggested adding the collection of wrong questions (29%) and providing explanations for those wrong choices (29%). They believe that this will help distinguish between the learning mode and assessment mode (14%) (Figure 4B).

## 4. Discussion

Apexification is a well-established technique to treat apical periodontitis and the pulp necrosis of immature permanent teeth [16]; however, these conditions cannot be simulated in vitro. Moreover, the traditional educational model is unable to meet dental students’ practical training needs; therefore, the VS learning platform attempts to make up for this deficiency by mimicking the entire clinical process. This allows students to gain the required knowledge prior to starting their clinic practice. This study mainly sought to achieve the optimal method of apexification learning and to productively apply a virtual simulation platform for apexification.

The complete steps of apexification designed for the virtual simulation learning platform are based on the guidelines on pulp therapy for primary and immature permanent teeth [20]. Although the inductive medicines for apexification have a series of options with the development of technology, calcium hydroxide (CH) and its modified calcium hydroxide pastes are classic and widely applied to reduce inflammation and induce an apical calcified barrier [21]. Vitapex (Neo Dental Inc., Tokyo, Japan) and Metapex (Meta Biomed Co. Ltd., Cheongju-si, Republic of Korea) belong to the category of calcium-hydroxide–iodoform–silicone-oil pastes and are mostly used in our pediatric department for apexification. The calcium-silicate-based materials for apexification, such as MTA, have exhibited superior advantages in reduced treatment time, are biocompatible, and provide a good sealing of the teeth apex [22]. However, CH and the modified CH paste for apexification have been demonstrated to possess similar treatment effects to those of MTA [23] and better potential to increase the root canal length [24]. Therefore, we mimic the shape and application method of Vitapex or Metapex in this VS learning platform instead of others.

Herein, the Exp group showed increased mastery of apexification compared with the Con group. Moreover, the knowledge of students about operative skills and the learning quality could be improved with the help of the VS learning platform, indicating an effective learning design that should be encouraged in the future. Previous studies have also revealed that simulated virtual techniques can enhance the attainment of operational skills and the sheer enjoyment of learning [13,25], in accordance with this study. In the Con group, watching the video on apexification could enhance the total points and part-C points in test 2 compared with those in test 1, suggesting that the use of videos in the pre-clinical training may have positive effects on the clinical practice in the internship. Consistently, Kenny et al. reported that viewing video clips showing pediatric local anesthetic administration can effectively improve students’ confidence when performing local anesthesia [26]. Gallardo et al. also found that using the videos in the flipped classroom of pediatric dentistry can encourage students to obtain more theoretical knowledge, which is beneficial for clinical practice [27]. Moreover, researchers compared the effects of two pedagogical platforms (Edpuzzle and Moodle 3.4) for flipped learning in the pre-clinical practices of pediatric dentistry. The results showed that the application of these platforms, especially Edpuzzle, can enhance the capacity of students in pediatric dentistry practices [28]. Therefore, the applications of videos and/or platforms possess great potential in pre-clinical training.

This study resulted in three practical and important benefits for the future pedagogy of apexification. Firstly, teachers can reference this research to modify new educational methods incorporating virtual simulation systems. Thus, dental students could acquire augmented apexification skills. In addition, the sharing and utilization of educational resources could be improved if students could preview and then review learning materials before and after class, respectively. Secondly, faculty-led teaching and supervision could be reduced using VS technology, freeing them for other tasks. However, such technology cannot completely supplant the mentoring role of instructors. It has been demonstrated that the most effective training still requires feedback from higher-status and more expert professionals [29]. However, VS systems are less costly over the long term [30]. Thirdly, the VS learning system can record students’ scores and mistakes automatically, and teachers can improve their teaching efficiency by analyzing the information provided by the VS platform.

Virtual reality and force feedback technologies are the main technologies included in the current dental virtual simulation system [31]. With the advancement of virtual simulation technologies, their application in dental education has been gradually increased in various fields, such as dental restoration, tooth and crown preparation, teeth implantation, caries removal, and so on [32]. Compared with the traditional teaching method, the VS technology in dental pedagogy possesses great advantages in eliminating the limitations of the environment, providing endless and timeless practicing opportunities, quick responses, simulated environment-based real experience, and improved educational quality [33,34]. Currently, VS is mainly recognized as a complementary tool for the traditional teaching mode, but some studies have suggested that the use of VS technology along with the traditional teaching method can be a favorable option for dental skill learning in students [8,35]. In the future, VS technologies will become more powerful with the development of big data, cloud computing, 5G, and deep learning technology [10]. The function of VS technology in the traditional education mode will be more diverse and individual.

Virtual simulation platforms are being developed rapidly, partially as a result of the valuable feedback from their users. The responses to the questionnaire survey provide valuable advice to improve this platform. According to the evaluations from students, a series of measures should be taken: (1) we must optimize the design of the toolbar to make it smoother; (2) adjust to the appropriate font size; (3) we must add clear instructions for each step; (4) we must add the function of returning to the previous step; (5) the whole system should be upgraded to improve time consumption and user satisfaction; (6) considering the widespread use of mobile phones, a mobile version of the system could be developed; and (7) the interface design should be more operable. Furthermore, the system of this VS platform can be optimized: (1) software operation can be smoother; (2) we must add more content; (3) live operation videos should be added to increase realism; (4) clear explanations about the choice of answers are needed to expand students’ knowledge; (5) it will be helpful to distinguish between the learning mode and assessment mode; (6) and we must develop the function of viewing the wrong-question set. This precious feedback points out the direction for our future study.

Nevertheless, this study has limitations. Notably, all the participants came from the same university, and thus might not represent the application situation of each dental school in China. Second, future research should assess the long-term effects of the VS learning system in terms of improving the proficiency of students in pre-clinical practice or the internship and the optimal time required to integrate this platform into the dental education curriculum. Third, the VS platform for apexification learning fails to simulate both the various anatomies and root canal systems of different teeth as well as different clinical scenarios. Fourth, there is a lack of a “test mode” on the VS platform to evaluate the manual skills of students, which is one of the drawbacks for this platform’s design and should be improved in the future.

Whether VS platforms could substitute the standard simulated head model experimental method as the major educational resource in the pre-clinical teaching curriculum is controversial. Further research is needed to provide corroborative evidence, as emphasized in a study on stereopsis in dentistry [36]. Previously, it was reported that virtual simulators showed fewer advantages for staff or students when carrying out cavity preparation in junior dental students; however, virtual reality simulation combined with conventional methods was shown to comprise the optimum approach [37]. Combining VS and jaw simulation in oral implant teaching has demonstrated better teaching efficiency [9]. Therefore, a combination of the VS technique and object teaching might be the best choice in the current pre-clinical teaching curriculum.

## 5. Conclusions

The present study established a simulated virtual learning platform for apexification and demonstrated this platform’s positive effect in improving the mastery of surgical knowledge and enhancing learning quality. Students can use the VS platform regardless of time and space constraints, thereby allowing them to develop their skills in a repeatable, safe, and noninvasive manner. The virtual simulation learning platform will promote educational resource optimization and sharing.

## Figures and Tables

**Table 1:** Comparison of Score T1.

S_T1_	Exp Group (*n* = 52)	Con Group (*n* = 47)	*p*-Value
Total points	9.0 (9.0, 10.0)	9.0 (9.0, 10.0)	0.594
Part A	3.0 (3.0, 4.0)	3.0 (3.0, 4.0)	0.968
Part B	3.0 (3.0, 4.0)	3.0 (2.0, 3.0)	0.146
Part C	3.0 (2.0, 4.0)	3.0 (3.0, 4.0)	0.499

**Table 2:** Comparison of Score T2.

S_T2_	Exp Group (*n* = 52)	Con Group (*n* = 47)	*p*-Value
Total points	11.0 (10.0, 11.0)	10.0 (9.0, 11.0)	<0.001 ***
Part A	3.5 (3.0, 4.0)	4.0 (3.0, 4.0)	0.470
Part B	4.0 (4.0, 4.0)	3.0 (2.0, 3.0)	<0.001 ***
Part C	4.0 (3.0, 4.0)	4.0 (3.0, 4.0)	0.802

*** *p* < 0.001.

**Table 3:** Comparison of S_T1_ and S_T2_ in the Con group.

Variable	S_T1_	S_T2_	*p*-Value
Total points	9.0 (9.0, 10.0)	10.0 (9.0, 11.0)	0.014 *
Part A	3.0 (3.0, 4.0)	4.0 (3.0, 4.0)	0.440
Part B	3.0 (2.0, 3.0)	3.0 (2.0, 3.0)	0.393
Part C	3.0 (3.0, 4.0)	4.0 (3.0, 4.0)	<0.001 ***

* *p* < 0.05, *** *p* < 0.001.

**Table 4:** Comparison of S_T1_ and S_T2_ in the Exp group.

Variable	S_T1_	S_T2_	*p*-Value
Total points	9.0 (9.0, 10.0)	11.0 (10.0, 11.0)	<0.001 ***
Part A	3.0 (3.0, 4.0)	3.5 (3.0, 4.0)	0.986
Part B	3.0 (3.0, 4.0)	4.0 (4.0, 4.0)	<0.001 ***
Part C	3.0 (2.0, 4.0)	4.0 (3.0, 4.0)	<0.001 ***

*** *p* < 0.001.

**Table 5:** Questionnaire analysis (*n* = 52).

Questions	Strongly Agree	Partially Agree	Disagree
I understand the apexification better with the help of the vs. learning platform.	63.46%	36.54%	0%
I master the key points and difficulties of apexification technique better with the help of the vs. learning platform.	63.46%	34.62%	1.92%
I have become familiar with the process of apexification with the help of the vs. learning platform.	80.77%	19.23%	0%
The vs. learning platform enhanced my enthusiasm to study apexification.	42.31%	51.92%	5.77%
The vs. learning platform is easy to operate.	57.69%	28.85%	13.46%
I like the vs. learning platform very much.	42.31%	51.92%	5.77%
The vs. learning platform is inconvenient and a waste of time.	7.69%	26.92%	65.38%

## Data Availability

Research data cannot be shared due to the requirements of the ethical approval granted.

## Appendix A

**Outline of questions in the virtual simulation platform**

The patient sought medical attention due to toothache and complained of spontaneous pain at night and occlusal pain. The occlusal surface of the second mandibular premolar was found to have circular marks and no caries was found. The patient experienced II degrees of percussion pain and I degree of looseness, with slight swelling in the apical area.

To clarify the diagnosis of this tooth, which examinations need to be performed? Please answer the pop-up question.

1. Choose the appropriate examination method for the affected tooth ( )
  - X-ray machine or X-ray image
  - Dental pulp vitality tester
  - Dental pulp sensory temperature tester
  - Percussion
  Explanations:
  A: Correct answer! Children and adolescents with traumatic teeth need to undergo X-ray examination to examine the degree of root development, periodontal ligament damage, and the presence of lesions around the apex. Imaging examination is an important basis for diagnosing young permanent teeth with periapical disease.
  B: Wrong answer! The development of the apical foramen of young permanent teeth is not complete, and the accuracy of pulp electrical vitality test is relatively low. In addition, the credibility of the response from children is relatively low. At the same time, there may be “pulp shock” in injured teeth. Therefore, clinical practice does not advocate using pulp electrical vitality test results to determine pulp status.
  C: Correct answer! Although the development of apical foramen in young permanent teeth is not yet complete, pulp vitality tests conducted in combination with medical history, intraoral examination, and X-ray examination can be used as a reference to determine the status of pulp.
  D: Correct answer! The status of the periapical periodontal ligament can be determined through percussion examination.
2. Facing the injured teeth, the doctor asked the child to take a dental X-ray, which showed that the apical foramen with large diameter was not closed, and there were shadows in the apical area. At this time, what is the most appropriate treatment method? ( )
  - Pulpotomy
  - Direct pulp capping
  - Apexification
  - Root canal therapy
  Explanations:
  A: Wrong answer! Based on clinical manifestations and examination results, the pulp of the affected tooth has necrotized and pulp preservation treatment cannot be chosen.
  B: Wrong answer! Based on clinical manifestations and examination results, the pulp of the affected tooth has necrotized and pulp preservation treatment cannot be chosen.
  C: Correct answer! Apexification is a classic operation for treating young permanent tooth with pulp and periapical disease. It is a method of preserving the apical pulp or depositing hard tissue around the apex with drugs such as calcium hydroxide on the basis of controlling infection, so as to promote the continued development of the root and the formation of the apex.
  D: Wrong answer!
3. Please perform moisture isolation operation, click ( ) to continue
  A. Rubber dam  B. Cotton roll
  Explanations:
  A: Correct answer! Rubber dams have a good moisture isolation effect and effectively reduce the possibility of pollution. Moreover, rubber dams increase operational safety and facilitate clear surgical field.
  B: Wrong answer! The moisture isolation effect of cotton rolls is poor, and they need to be constantly replaced, which is not conducive to aseptic treatment.
4. Open the pulp chamber along the puncture site to prepare the channel. Please select a tool ( )
  A. Fast dental handpiece  B. Slow dental handpiece
  Explanations:
  A: Correct answer! The fast dental handpiece can efficiently remove the wall detritus and most of the bottom detritus, and prepare the necessary cave shape.
  B: Wrong answer!
5. Please choose the appropriate bur for opening of the pulp chamber ( )
  A. Fissure bur  B. Round bur  C. Inverted bur
  Explanations:
  A: Wrong answer!
  B: Correct answer! The fissure bur can open the pulp and prepare a straight-line channel, and the round bur can lift and remove the top of the pulp chamber.
  C: Wrong answer!
6. Choose the appropriate root canal irrigation solution ( )
  A. Saline+3% hydrogen peroxide    B. 5.25% sodium hypochlorite
  C. 17% EDTA solution           D. 7.5% povidone iodine
  Explanations:
  A: Correct answer! Adequate irrigation and effective control of inflammation are important steps in apexification.
  B: Wrong answer! Excessive concentration of sodium hypochlorite has a certain toxic effect, which can stimulate and damage the periapical tissue.
  C: Wrong answer! EDTA solution needs to be combined with sodium hypochlorite to achieve good cleaning effect.
  D: Wrong answer! 7.5% povidone iodine can easily cause tooth discoloration.
7. Select nickel-titanium (NiTi) instruments with appropriate taper for root canal cleaning and preparation ( )
  A. #8 NiTi file  B. #15 NiTi file  C. #30 NiTi file
  Explanations:
  A: Wrong answer! The root canal of young permanent teeth is relatively large, and NiTi files with too small taper cannot effectively clean and prepare the root canal, and are prone to extending beyond the apical foramen.
  B: Wrong answer! The root canal of young permanent teeth is relatively large, and NiTi files with too small taper cannot effectively clean and prepare the root canal, and are prone to extending beyond the apical foramen.
  C: Correct answer! Young permanent teeth have larger root canals, and the selection of large taper NiTi files can effectively clean and prepare the root canal.
8. Select appropriate materials for apexification ( )
  A. Vitapex or Metapex  B. Gutta-percha  C. Light cured calcium hydroxide
  D. Handmade calcium hydroxide
  Explanations:
  A: Correct answer! Vitapex or Metapex is a manufactured calcium hydroxide oil-based product with a certain viscosity, which is easy to place at the root apex, slows down the rate of calcium hydroxide decomposition, plays a sustained antibacterial role, and promotes sealing of root apex.
  B: Wrong answer! Gutta-percha is a non-absorbable root filling material and cannot be used when the apical foramen of young permanent teeth is not closed.
  C: Wrong answer! Light curing calcium hydroxide cannot be used for root canal disinfection.
  D: Wrong answer! The disinfection effect of handmade calcium hydroxide only lasts for about 2 weeks. Frequent dressing changes will increase the stimulation of the affected root apex, and repeated opening of the pulp cavity will also increase the risk of reinfection. Moreover, handmade calcium hydroxide is difficult to fill the root apex tightly.
9. Please select the appropriate temporary sealing material ( )
  A. Zinc oxide (ZOE)  B. Glass ionomer
  Explanations:
  A: Wrong answer! ZOE has a good sealing effect within 1 week, and the coronal leakage will increase with the extension of the temporary sealing time. It is suitable for short-term treatment, and it is not recommended for treatment requiring temporary sealing for more than 2 weeks. Moreover, eugenol can affect the polymerization of the resin, and manual modulation may lead to unstable performance.
  B: Correct answer! Glass ionomers have good edge sealing properties, reduce micro leakage, and release fluoride ions that can induce partial softened dentin remineralization, which is suitable for treatment with a long observation period such as apexification and pulpotomy.
10. After completing apexification, please choose the right time for shape repair ( )
  A. After apexification  B. After apical closure
  Explanations:
  A: Correct answer! After the child has no conscious symptoms, the appearance repair of teeth as soon as possible can restore the aesthetic and chewing function in time, and maintain the gap.
  B: Wrong answer! Long-term dental defects will reduce the aesthetic and chewing function of children, lead to psychological disorders, and may cause loss of space.
11. The appropriate timing for permanent root canal treatment of affected teeth after apexification is ( )
  A. When the root apex of the affected tooth is closed  B. The patient is 12 years old
  C. The patient is 18 years old
  Explanations:
  A: Correct answer! When the apical foramen of the affected tooth is closed or the apical barrier is formed, permanent root canal treatment can be performed.
  B: Wrong answer! There are significant individual differences in the degree of tooth development, especially in the development of traumatic teeth. It is impossible to determine the treatment time of affected teeth based on age alone.
  C: Wrong answer! There are significant individual differences in the degree of tooth development, especially in the development of traumatic teeth. It is impossible to determine the treatment time of affected teeth based on age alone.

## Appendix B

**Test 1**

**Please choose the right answer from the following one-choice questions:**

**Part A**

1. Apexification is suitable for ( )
  - Deciduous teeth
  - Permanent teeth with fully developed tooth roots
  - Young permanent teeth with pulp necrosis or concurrent periapical inflammation
2. Which of the following is not a success criterion for apexification? ( )
  - The root apex continues to develop, the root canal becomes narrow, and the root apex is closed.
  - The root canal has no change but the root apex is closed.
  - There is a trace of colored liquid discharged from the root canal.
  - No development was observed, and hard tissue barrier formation was detected in the root canal.
  - A calcification barrier is formed at 1/3 of the root end.
3. What is the incorrect description about apexification? ( )
  - The infected material in the root canal is removed and induced by drugs to promote root development and apical closure.
  - Calcium hydroxide preparations are commonly used inducers.
  - It is suitable for young permanent teeth with severe pulp lesions or concurrent periodontitis before the root development is complete.
  - It is suitable for the tooth with residual living pulp at the root end, or the dental papilla has not been damaged.
  - Successful apexification means that the root continues to develop and the apex is formed.
4. A 7 years old boy had spontaneous pain in the first lower right permanent molar. He was unable to sleep for the past two days. Examination revealed deep caries and exposed pulp on the occlusal surface. Discomfort during probing (+) and percussion. No redness or swelling in the gums. What treatment should be taken? ( )
  - Root canal treatment
  - Pulp mummification
  - Apexification
  - Filling after oral anti-inflammatory medication
  - Direct pulp capping

**Part B**

5. What is the incorrect statement about the operation steps of apexification? ( )
  - Teeth with acute symptoms should first undergo emergency treatment, open the root canal, and continue treatment after the acute inflammations disappear.
  - The root canal preparation was mainly mechanical preparation, supplemented by chemical preparation.
  - X-rays are routinely taken before treatment.
  - The position and size of pulp opening should be such that the instrument enters the root canal in a straight line as much as possible.
6. When performing apexification, the stopping point of the operation should be located at ( ).
  A. The end of the root tip        B. 1 mm above the end of the root tip
  C. 0.5 mm above the end of the root tip  D. 2 mm above the end of the root tip
7. The commonly used irrigation solution for root canal preparation during apexification does not include ( )
  A. 17%EDTA      B. 3% hydrogen peroxide
  C. physiological saline  D. 1.5% sodium hypochlorite
8. Which of the following drugs should not be used as root canal disinfectants during apexification? ( )
  A. Pomegranate oil  B. Glutaraldehyde  C. Camphor phenol  D. Antibiotic paste

**Part C**

9. The statement about calcium hydroxide as apexification inducer is incorrect ( )
  - The induction effect of calcium hydroxide originates from its strong alkalinity and the combined effect of calcium ions.
  - Calcium hydroxide is currently the preferred drug for apexification.
  - Calcium hydroxide water paste is easy to fill the root apex tightly.
  - Calcium hydroxide is a paste that is easily absorbed by inflammatory tissues.
10. Which of the following statements is incorrect during apexification re-examination? ( )
  - Check if the filling material is intact.
  - Replace calcium hydroxide every time when the re-examination is conducted.
  - X-ray examination should be taken.
  - Observe the periapical condition and apical formation status.
11. The tissues on which apexification depends do not include ( )
  A. Periodontal ligament       B. Epithelial root sheath
  C. Residual living pulp at the apex  D. Dental papilla at the apex
12. Which of the following is NOT a disadvantage of apexification compared with apical barrier surgery? ( )
  A. Longer treatment cycle     B. Significantly lower success rate
  C. Increased risk of root fracture  D. Higher risk of reinfection in the root canal

## Appendix C

**Test 2**

**Please choose the right answer from the following one-choice questions:**

**Part A**

1. Female, 8 years old, presents 1 week after anterior tooth trauma. Right upper central incisor crown fracture 2/3, subgingival 1 mm on the mesial side. There is no pain during probing at the exposed pulp, and percussion (+). The tooth shows dark red bleeding and degree I looseness. X-ray shows no root fracture, and the root apex appears as a trumpet shaped opening. Which is the appropriate treatment method choice? ( )
  - Calcium hydroxide pulpotomy
  - Formaldehyde cresol pulpotomy
  - Apexification
  - Root canal therapy
2. How often should follow-up be conducted after apexification? ( )
  A. 1–3 months  B. 3–6 months  C. 9–12 months  D. 6–9 months  E. 12 months
3. What is the correct timing for permanent root canal filling after apexification? ( )
  A. 12 years old                B. When the apical calcification barrier begins to form
  C. When the apical calcification barrier forms  D. When there is calcified tissue deposition
4. Which of the following is considered as ineffective treatment according to the evaluation criteria for apexification? ( )
  - Failure to extend the tooth root or no reduction or disappearance of periapical lesions
  - Periapical lesions disappear, roots elongate, root canal shrink, apical formation or root closure occurs
  - Periapical lesions disappear, roots elongate, incomplete or highly irregular apical formation

**Part B**

5. When performing apexification, the method to determine the working length of the root canal is ( )
  A. Root canal length measuring instrument  B. Referring to the preoperative X-ray
  C. Determining the location of apical stenosis by hand feeling
6. Which of the following statements is incorrect during root canal preparation for apexification? ( )
  - Whether it is mechanical preparation or chemical flushing, attention should be paid to avoiding damage to the apical dental papilla or epithelial root sheath.
  - Due to the large size of the apical foramen of young permanent teeth, the root canal instruments can be slightly extended beyond the apical foramen during mechanical preparation.
  - Be careful not to apply pressure during rinsing to avoid pushing infectious substances out of the root apex.
  - When performing root canal preparation, the movements should be as gentle as possible.
7. The material that is not suitable for apexification is ( )
  A. Vitapex  B. Metapex  C. Photocured calcium hydroxide  D. MTA
8. The appropriate timing for shape restoration after completing apexification is ( )
  A. After completing apexification     B. After apical closure
  C. Formation of apical calcification barrier  D. After root canal treatment

**Part C**

9. Which of the following is a possible treatment option after failure of apexification? ( )
  A. Apical barrier  B. revascularization  C. pulpotomy
10. On X-rays, a calcified barrier can be seen at one-third of the root apex. According to Frank classification, which type of root development after apexification is this? ( )
  A. A-type  B. B-type  C. C-type  D. D-type
11. What is the wrong statement about the function of epithelial root sheath in apexification? ( )
  - Epithelial root sheath can prevent periodontal ligament cells from growing into the root canal.
  - There are a large number of undifferentiated mesenchymal cells in the epithelial root sheath, which can further differentiate into hard tissue.
  - If the Hertwig epithelial root sheath is completely destroyed, it will lead to the cessation of normal tooth root development and also indicates the end of hard tissue deposition in the apical area of the root.
  - During treatment, it is important to preserve the activity of the epithelial root sheath as much as possible.
12. Which of the following is not a factor in determining the overall duration of apexification? ( )
  A. Frequency of dressing change  B. Degree of periapical inflammation
  C. patient’s physical condition   D. The original length of the root of the tooth
